# MultiPro: DDA-PASEF and diaPASEF acquired cell line proteomic datasets with deliberate batch effects

**DOI:** 10.1038/s41597-023-02779-8

**Published:** 2023-12-02

**Authors:** He Wang, Kai Peng Lim, Weijia Kong, Huanhuan Gao, Bertrand Jern Han Wong, Ser Xian Phua, Tiannan Guo, Wilson Wen Bin Goh

**Affiliations:** 1 Lee Kong Chian School of Medicine, Nanyang Technological University, Singapore, 308232 Singapore; 2 School of Biological Sciences, Nanyang Technological University, Singapore, 637551 Singapore; 3 Westlake Center for Intelligent Proteomics, Westlake Laboratory of Life Sciences and Biomedicine, Hangzhou, Zhejiang Province 310030 China; 4 Key Laboratory of Structural Biology of Zhejiang Province, School of Life Sciences, Westlake University, Hangzhou, Zhejiang Province 310030 China; 5 Research Center for Industries of the Future, Westlake University, 600 Dunyu Road, Hangzhou, Zhejiang 310030 China; 6 Center for Biomedical Informatics, Nanyang Technological University, Singapore, 636921 Singapore

**Keywords:** Data publication and archiving, Protein databases

## Abstract

Mass spectrometry-based proteomics plays a critical role in current biological and clinical research. Technical issues like data integration, missing value imputation, batch effect correction and the exploration of inter-connections amongst these technical issues, can produce errors but are not well studied. Although proteomic technologies have improved significantly in recent years, this alone cannot resolve these issues. What is needed are better algorithms and data processing knowledge. But to obtain these, we need appropriate proteomics datasets for exploration, investigation, and benchmarking. To meet this need, we developed MultiPro (Multi-purpose Proteome Resource), a resource comprising four comprehensive large-scale proteomics datasets with deliberate batch effects using the latest parallel accumulation-serial fragmentation in both Data-Dependent Acquisition (DDA) and Data Independent Acquisition (DIA) modes. Each dataset contains a balanced two-class design based on well-characterized and widely studied cell lines (A549 vs K562 or HCC1806 vs HS578T) with 48 or 36 biological and technical replicates altogether, allowing for investigation of a multitude of technical issues. These datasets allow for investigation of inter-connections between class and batch factors, or to develop approaches to compare and integrate data from DDA and DIA platforms.

## Background & Summary

Mass spectrometry (MS)-based proteomics has important applications for biomedical research in the areas of phenotype correlation and characterization^1^, biomarker and drug discovery^2,3^, and therapeutics^4^. Proteomic data and technologies serve as a vital corroboration layer over mainstream gene expression “-omics” platforms. Unlike the gene layer which can only suggest or imply functionalities, proteomics provide direct information on the proteins, which are the functional agents, providing direct information on mechanisms and phenotypes.

Although powerful, proteomic data is challenging to generate, use and study. Proteomics is not simply a single technology but comes in many flavours and platforms. For example, proteomics can be pursued via different data acquisition modes, e.g., data-dependent acquisition (DDA) and the more recent data-independent acquisition (DIA) coupled with various ion-capture configurations, liquid chromatography (LC) systems and quantitation strategies (e.g., labelled and label-free). This diversity creates challenges for cross-platform/cross-mode data integration^5^. The non-amplifiable nature of proteins and instrument resolution limits lead towards missing values, especially amongst low-abundant proteins^6^. Finally, instrument throughput issues mean samples often need to be split (batched) across various machines and laboratories. This can produce challenging technical biases known as “batch effects”^7–9^.

The issues of technological diversity, missing values and batch effects can confound true biological signals, reduce reliability of downstream statistical analysis, and lead towards research outcome irreproducibility^5,6,8^. Moreover, it is naïve to think of these issues as independent: Recent works have demonstrated co-dependence and interactions between these issues; e.g., between missing value imputation (MVI) and batch effect correction (BEC)^10^. We know that when MVI algorithms are applied across entire batch factors, the imputed values no longer maintain the batch factor structures and cannot be corrected by batch effect correction algorithms (BECAs). This leads towards higher variances per sample, and ultimately, higher false positive/negative rates. It is insufficient to simply apply general algorithms (e.g., MissForest and KNNimpute for MVI)^11,12^ or borrow established methods from similar domains (such as ComBat for BEC, which is originally from microarray data)^13^. It may be better to develop specific algorithms for the platform. But to do this, we will need appropriate benchmark or evaluation datasets.

Benchmark datasets are vital for algorithm development, evaluation and furthering our understanding of technical and biological phenomena. In MVI, a wide range of popular MVI methods have been benchmarked against a battery of public proteomics datasets representing diverse tissue/sample types^14,15^. In BEC, proBatch was recently developed as a methodological framework for resolving batch effects in proteomics data. proBatch was accompanied with good quality benchmark datasets to empower community efforts on new method development^7^. BIRCH is an automated R Shiny tool to facilitate diagnosis and correction of batch effects, with well-characterised batch affected proteomics datasets^16^.

These advances demonstrate that dealing with technical biases are important. However, gaps remain. The works mentioned above were based on datasets not originally designed or intended for benchmarking. These were meant for answering specific research questions, which also means that biological heterogeneity is present as a confounder. Numerous technical replicates, predicated on the same biological sample, are invaluable for studying technical variation due to instrument and acquisition mode. But the datasets mentioned above mostly contained biological class information and are unlikely to have sufficient technical replicates to study platform-specific issues. Hence, they may be unsuitable for deep diving into technical effect investigations.

To illustrate some examples, we turn first to MVI. In MVI benchmark datasets, previous works mainly focused on DDA datasets with a small sample size (less than 20), which is not representative enough for various missing structures and potential confounding between MVI and BEC, especially in current mainstream DIA-based large-scale proteomic datasets^14,15^. For BEC, two datasets in proBatch^7^ suffer from MS signal drift (should be corrected by normalization algorithm), which introduces additional interferences in benchmarking BECAs. And these datasets, generated for biomedical problems, comprise mostly biological replicates from different individuals like patients or mice, resulting in higher and unnecessary heterogeneity^7,16^.

Furthermore, there are very few cross-platform/cross-mode datasets, e.g., running the same samples on both DDA and DIA. Most clinical proteomic datasets were only acquired through one platform. Specifically, in proBatch^7^, three datasets were solely acquired through DIA and the other two from label-free DDA or TMT, limiting their usefulness as benchmark data for investigating, comparing, and integrating across proteomics platforms. Finally, there is a lack of benchmark datasets derived from new promising technologies such as PASEF (Parallel Accumulation-Serial Fragmentation), which has been developed through Trapped Ion Mobility Spectrometry Time of Flight (timsTOF) mass spectrometry to provide significant gains in sensitivity, coverage and reproducibility^17^.

To address the above issues, we present MultiPro (Multi-purpose Proteome Resource), which comprises a suite of four comprehensive large-scale proteomic datasets with deliberate design. The samples were generated in DDA-PASEF^18^ or diaPASEF^19^ mode using standard data processing workflows^20^ (see next section for details). MultiPro contains a balanced two-class design based on well-characterized cell lines (A549 vs K562 or HCC1806 vs HS578T), with controlled batch effects established from two different MS instruments and 48 or 36 biological and technical replicates altogether in each dataset. This design has the following use cases: Direct DDA-PASEF and diaPASEF pairings per sample allow for meaningful validation of predicted missing proteins/values. These pairings are also useful as validations when attempting to use generative AI to transform data from one platform type to another, allowing for advanced applications to improve cross-platform inter-operability and facilitating data integration. Given the present batch factors, investigators can also evaluate BECAs in terms of the consistency of returned differential proteins, or the consistency of results across batch/class pairs. Finally, MultiPro could interact with other published datasets^21,22^ which also quantified cell lines same as ours (A549, K562, HCC1806 and HS578T) but using diverse data generation paradigms across various time periods, to mimic different kinds of batch effects (e.g., same instrument but different days, instruments across different labs, different LC columns) and benchmark BECAs thoroughly.

## Methods

### Experimental design

**Fig. 1:**
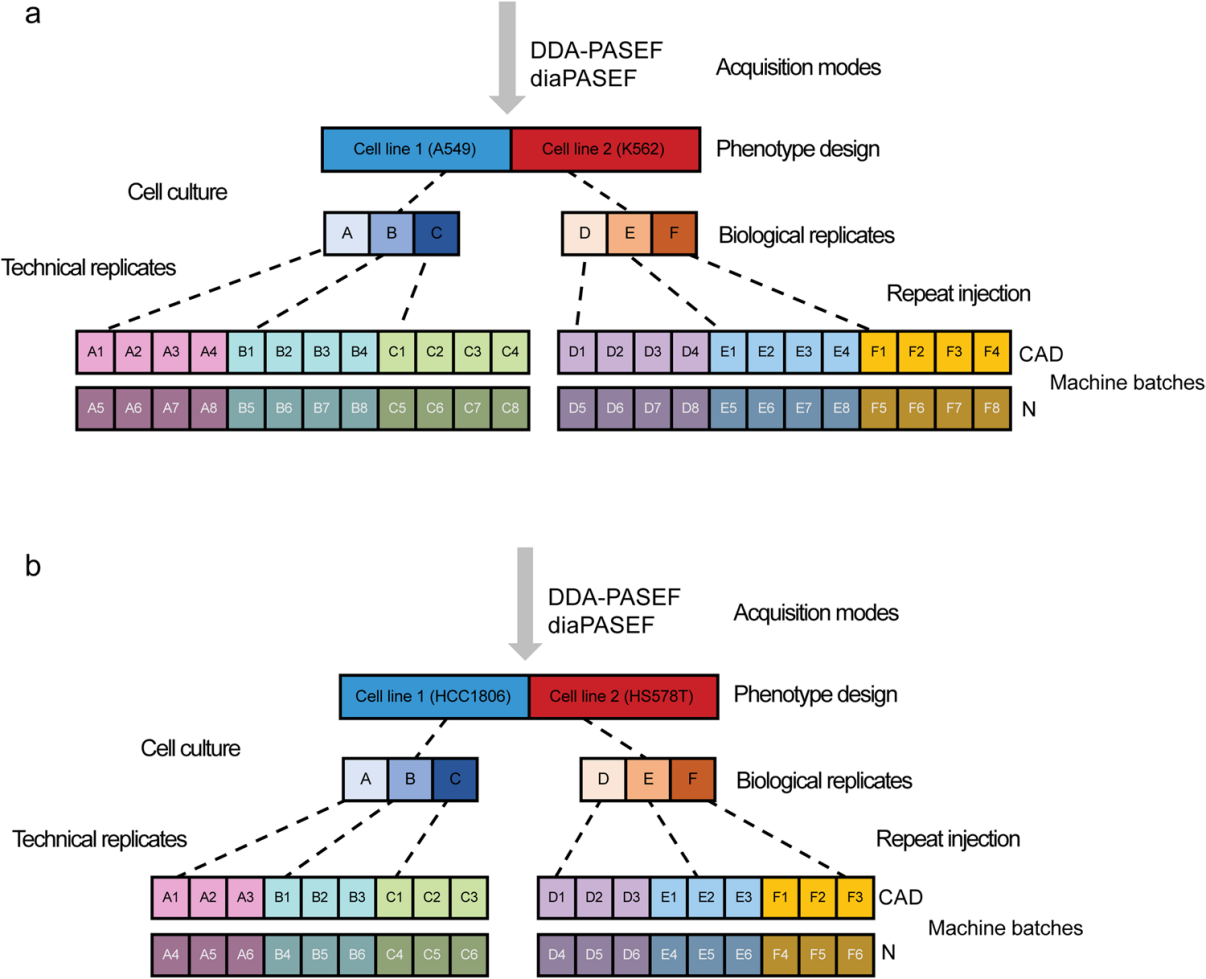
Experimental design of A549/K562 and HCC1806/HS578T cell line pairs acquired through timsTOF Pro mass spectrometer with DDA-PASEF and diaPASEF modes. (**a**) A549/K562 experimental design. For each cell line, there are three biological replicates, and each biological replicate was replicated four times as technical replicates, which were further quantified in parallel by two timsTOF Pro instruments (named CAD and N) from the same laboratory. (**b**) HCC1806/HS578T experimental design. It is all the same but with three technical replicates generated for each biological replicate.

The first pair of datasets were acquired both through timsTOF Pro mass spectrometer with DDA-PASEF or diaPASEF modes along well-characterized and widely studied cell lines pair^23–25^: A549 (lung carcinoma epithelial cells) and K562 (bone marrow lymphoblast cells from a chronic myelogenous leukaemia patient) (Fig. 1a). Each dataset is characterised by three biological replicates corresponding to each cell line, with each biological replicate in turn, replicated four times as technical replicates. To generate batch effects, two timsTOF Pro machines (named CAD and N) from the same laboratory were used to quantify each technical replicate in parallel. In total, each dataset (DDA-PASEF/diaPASEF) contains 48 samples, divided equally between two batches (Batch 1 acquired by CAD and Batch 2 by N), with 12 samples from A549 and 12 from K562 in each batch.

The study design is similar in the remaining pair of datasets (Fig. 1b), containing phenotype classes from two well-established cell lines: HCC1806 (breast carcinoma epithelial cells^26,27^) and HS578T (also breast carcinoma epithelial cells^28,29^). Instead, each biological replicate was subjected to three technical replicates. In total, 36 samples were quantified using each acquisition mode, with 18 samples in Batch 1 (CAD) and 18 samples in Batch 2 (N), and 9 samples derived from HCC1806 and 9 from HS578T in each batch.

### Cell culture and sample preparation

The A549 and K562 cell lines (National Collection of Authenticated Cell Cultures, China) were cultured in Ham’s F-12K medium (Invitrogen, USA) and Iscove’s modified Dulbecco’s medium (Invitrogen, USA) separately, supplemented with 10% fetal bovine serum and 1% penicillin-streptomycin. Cells were split into three dishes as biological replicates, then washed with phosphate-buffered saline and collected by centrifugation three times.

The cells for each biological replicate were then subjected to denaturation, reduction, alkylation and digestion referring to the accelerated pressure cycling technology (PCT) workflow^20,30^. Briefly, each sample was transferred into a PCT-MicroTube (Pressure Biosciences Inc., USA) with 30 μL lysis buffer (6 M urea and 2 M thiourea from Sigma-Aldrich, Germany), 20 mM tris (2-carboxyethyl) phosphine (TCEP, Adamas-beta, China), and 40 mM iodoacetamide (IAA, Sigma-Aldrich, Germany) added. The PCT scheme in this step involved 90 oscillating cycles, each with 45,000 psi for 30 s and ambient pressure for 10 s at 30 °C using the Barocycler (Pressure Biosciences Inc., USA). Next, the processed proteins were digested by a mixture of trypsin and Lys-C (Hualishi Tech. Ltd, China) with an enzyme-to-substrate ratio of 1:20 and 1:80, respectively, using the PCT scheme consisting of 120 cycles at 30 °C, with 50 s of high pressure at 20,000 psi and 10 s of ambient pressure for each cycle. Trifluoroacetic acid (TFA, Thermo Fisher Scientific, USA) was added to the solution to a final concentration of 1% to stop enzymatic digestion. Subsequently, peptides were desalted by the SOLAμ HRP 96-well plate (Thermo Fisher Scientific, USA) and dried by a SpeedVac (Thermo Fisher Scientific, USA) based on the manufacturer’s protocol. The peptide concentration was measured using a ScanDrop^2^ spectrophotometer (Analytik Jena, Germany).

The HCC1806 and HS578T cell lines (American Type Culture Collection, ATCC, USA) were cultured separately in RPMI-1640 (Cytiva, USA) and Dulbecco’s modified Eagle medium (Cytiva, USA) with 10% fetal bovine serum and 1% penicillin-streptomycin. The procedures for cell culture and peptide extraction were the same as those previously described for the A549/K562 cell lines.

### LC-MS/MS in DDA-PASEF and diaPASEF modes

For each biological replicate, the protein digest samples were split into two vials (one for each timsTOF Pro machine), each vial included enough peptides for four/three injections to derive four/three technical replicates. Then the injected peptides were analysed using a nanoElute ultra-high-performance liquid chromatography (UHPLC) system (Bruker Daltonics, Germany). Around 200 ng peptides were reconstituted in buffer A (0.1% formic acid in water (0.1% FA/water), MS grade, Thermo Fisher Scientific, USA), trapped by 5 mm × 300 μm ID trap column (Thermo Fisher Scientific, USA) packed with 5 μm 100 Å C18 aqua in 0.1% FA/water and then separated at a flow rate of 300 nL/min using a 60-minute gradient on a 15 cm analytical column (75 μm ID, 1.9 μm, C18 beads, homemade). The mobile phase B consisted of 0.1% formic acid in acetonitrile (ACN), and its gradient comprised three linear segments: from 5 to 27% in 50 min, from 27 to 40% in 10 min, and from 40 to 80% in 2 min, with an additional 3-minute 80% sustain for analytical column washing. All separation processes were performed in an integrated toaster column oven at 50 °C.

The LC was coupled to a timsTOF Pro mass spectrometer (Bruker Daltonics, Germany) equipped with a CaptiveSpray nano-electrospray ion source. DDA was performed in PASEF^18^ mode with 10 PASEF MS/MS scans. The capillary voltage was set to 1500 V and the spectra were acquired in the range of m/z from 100 to 1700 Th with an ion mobility range (1/K0) from 0.6 to 1.6 Vs/cm^2^. The ramp and accumulation time were set to 100 ms to achieve a duty cycle close to 100% and a total cycle time of 1.17 s. The collision energy was ramped linearly as a function of mobility from 59 eV at 1/K0 = 1.6 Vs/cm^2^ to 20 eV at 1/K0 = 0.6 Vs/cm^2^. Precursors with charge state from 0 to 5 (If the charge state of a peptide precursor is 0, it indicates that the isotope ion was not detected for that peptide precursor) were selected with the target value of 20,000 and intensity threshold of 2500. Any precursors that reached the target value in arbitrary units were dynamically excluded for 0.4 min.

For diaPASEF acquisition^19^, the capillary voltage was set to 1400 V. The MS1 and MS2 spectra were obtained over a mass-to-charge (m/z) range of 100–1700 Th, with an ion mobility range (1/K0) of 0.7–1.3 Vs/cm^2^. The other setting was the same as DDA-PASEF mode. Additionally, a 28 Th isolation window was defined, ranging from 384 to 1059 Th (The detailed setting of the diaPASEF isolation windows was shown in Supplementary Table 1).

### MS data analysis

#### DDA-PASEF by FragPipe

DDA-PASEF raw data was processed using the FragPipe platform^31^ (version 19.1), integrating tools such as MSFragger^32,33^ (version 3.7), IonQuant^34^ (version 1.8.10), Philosopher^35^ (version 4.8.0), and Python (version 3.9.12). The database search was directed against a FASTA file comprising 20,437 protein entries, which included 20,389 human proteins from UniProt and 48 supplemented contamination proteins from FragPipe, and an equal number of decoy sequences added by FragPipe.

The analysis was set to the “LFQ-MBR (Label-Free Quantification and Match-Between-Runs)” workflow, utilizing the “IM-MS (Ion Mobility, timsTOF only)” input mode. Peak matching was characterized by a precursor tolerance of −20 to 20 PPM and a fragment mass tolerance of 20 PPM. We engaged functions like deisotoping, mass calibration, and parameter optimization. Digestion settings ensured usage of the “stricttrypsin” enzyme, focusing on fully tryptic peptides (with up to 2 missed cleavages), within a peptide length range of 7–50 and mass range of 500–5000. The modifications were categorized with methionine oxidation and protein N-terminal acetylation as variable, while carbamidomethylation in cysteine was deemed fixed. Constraints were set for a maximum of 3 variable modifications on a peptide and 5 modification combinations.

For validation, we incorporated Percolator^36^ with a 0.5 minimal probability for peptide-spectrum match (PSM) validation and enabled MSBooster^37^ for rescoring using deep learning-aided retention time and spectra prediction. A false discovery rate (FDR) of 1% was applied at both the PSM and protein levels (sequential filtering), utilizing the corresponding decoy sequences for FDR estimation. The quantification stage witnessed normalization of intensities across runs, adopting the peptide-protein uniqueness option as “unique plus razor”. Specifications for feature detection mandated a minimum of 3 scans and 2 isotopic peaks. Tolerances for peak tracing were 10 PPM (m/z), 0.4 minutes (retention time), and 0.05 1/K0 (ion mobility). Additionally, MaxLFQ^38^ intensity was integrated into quantification results, necessitating a minimum of 2 ions for protein quantification. Match-between-runs was activated, aligning with FDR 1% at ion, peptide and protein levels, retention time tolerance 1 minute, and ion mobility tolerance 0.05 1/K0, with constraints on minimal correlation and top runs set to 0 and 100 respectively. Unless specified, default settings were maintained for all parameters.

#### diaPASEF by DIANN

The diaPASEF raw data was processed using DIA-NN^39,40^ (version 1.8.1) in a library-free search mode. This analysis utilized a FASTA file encompassing 20,368 human protein entries and 286 contamination proteins. The precursor ions were generated from FASTA in silico digest with deep learning-based spectra, retention times (RTs), and ion mobilities (IMs) prediction. The DIA runs then facilitated the creation of a spectral library based on protein IDs, RTs, and IM profiling strategies. This library was subsequently employed to re-analyze the DIA runs.

Several parameters were optimized automatically from the first experimental run, including MS1 and MS2 mass accuracies and the scan window. The proteolytic enzyme Trypsin/P was selected, allowing for up to one missed cleavage. Modification settings incorporated the enablement of N-terminal methionine excision and the specification of cysteine carbamidomethylation as a fixed modification. Variable modifications were restricted to none. Parameter specifics include peptide lengths ranging from 7–30, precursor charges from 1–4, precursor m/z from 300–1800, and fragment ion m/z from 200–1800.

Further enhancements included enabling match-between-runs, isotopologues, heuristic protein inference, and no shared spectra. The neural network classifier operated in single-pass mode, while the quantification strategy was denoted as robust LC for high precision. In all instances, protein isoforms were consistently grouped according to their FASTA protein names. To facilitate cross-run normalization, an RT-dependent normalization strategy was adopted. The FDR was specified at 1% for both precursors and protein groups. All other parameters remained at their default settings.

## Data Records

### A549/K562 datasets

#### Repository and identifier

The A549/K562 proteomics datasets^41^ (instrument raw files, FASTA files and database search results) have been deposited to the ProteomeXchange Consortium via the PRIDE partner repository (https://www.ebi.ac.uk/pride/) with the dataset identifier PXD041421.

#### Directory structure and contents

1. DDA-PASEF: 48 MS raw files (Bruker .d format, annotated by RAW), 1 compressed original database search results folder (annotated by SEARCH) including all the reports, log, annotations, and intermediate results, 1 compressed folder also annotated by SEARCH including 48 standardized identification files (mzIdentML format) and 1 FASTA file (annotate by FASTA).
2. diaPASEF: 48 MS raw files (Bruker .d format, annotated by RAW), 1 FASTA file (annotated by FASTA), and 1 compressed original database search results folder (annotated by SEARCH) including all the reports, generated libraries, log, annotations, and intermediate results.

### HCC1806/HS578T datasets

#### Repository and identifier

The HCC1806/HS578T proteomics datasets^42^ (instrument raw files, FASTA files and database search results) have been deposited to the ProteomeXchange Consortium via the PRIDE partner repository (https://www.ebi.ac.uk/pride/) with the dataset identifier PXD041391.

#### Directory structure and contents

1. DDA-PASEF: 36 MS raw files (Bruker .d format, annotated by RAW), 1 compressed original database search results folder (annotated by SEARCH) including all the reports, log, annotations, and intermediate results, 1 compressed folder also annotated by SEARCH including 36 standardized identification files (mzIdentML format) and 1 FASTA file (annotate by FASTA).
2. diaPASEF: 36 MS raw files (Bruker .d format, annotated by RAW), 1 FASTA file (annotated by FASTA), and 1 compressed original database search results folder (annotated by SEARCH) including all the reports, generated libraries, log, annotations, and intermediate results.

## Technical Validation

### Proteome identification

The workflow outlined above was employed to develop MultiPro with a balanced two-class design (cell lines: A549 vs K562 or HCC1806 vs HS578T) using either DDA-PASEF or diaPASEF mode. We identified a high number of peptides/precursors and proteins/protein groups from each dataset (Table 1). Overall, 80520/67095 peptides were identified from 7290/7197 proteins in two DDA-PASEF datasets whereas 133802/123143 precursors from 8813/8895 protein groups in the diaPASEF datasets. Delving into the specifics, a singular sample from two DDA-PASEF datasets yielded averages of 77802/76467 PSMs (Interquartile range (IQR): 71232 - 84330/74516 - 79989), 38208/37019 peptides (IQR: 35745 - 39524/36150 - 38213) and 5275/5566 proteins (IQR: 5070 - 5450/5464 - 5666). In contrast, the DIA-acquired datasets revealed 104738/101308 precursors (IQR: 98905 - 112020/99147 - 103356) and 7994/8282 protein groups (IQR: 7902 - 8075/8271 - 8298) on average per sample (Specific identification results for each sample and other statistics were summarized in Supplementary Table 2). When examining protein coverages (Supplementary Fig. 1), the DDA-PASEF datasets presented means of 22.2% and 20.7% (IQR: 6.5% - 34.5%/6.3% - 31.1%). The diaPASEF counterparts, however, showcased averages of 27.0% and 25.8% with IQR from 11.5%/11.0% to 40%/37.7% (Detailed statistics were shown in Supplement Table 3). Therefore, compared to DDA, DIA paradigm identified more than 20% higher number of proteins and had better protein coverage, indicating higher sensitivity and robustness of DIA method.

**Table 1:** The identification results of A549/K562 and HCC1806/HS578T cell line pairs acquired through timsTOF Pro mass spectrometer with DDA-PASEF and diaPASEF modes.

Datasets	Sample size	#PSMs	#Peptides	#Proteins
Datasets	Sample size	#Precursors	#Protein groups	
A549/K562 DDA-PASEF	48	77802	38208 (80520)	5275 (7290)
HCC1806/HS578T DDA-PASEF	36	76467	37019 (67095)	5566 (7197)
A549/K562 diaPASEF	48	104738 (133802)	7994 (8813)	
HCC1806/HS578T diaPASEF	36	101308 (123143)	8282 (8895)	

# the average number of; (): the number in parenthesis denotes the overall identification number from all the samples.

To underscore the reliability of our identification results, we have collated the identification scores from these four datasets. For the DDA-PASEF datasets analyzed using FragPipe, the confidence scores are gauged by the PeptideProphet probability^43^ at both PSM and peptide levels. At the protein level, we employed the top peptide probability, which represents the highest probability among the supporting peptides. For both metrics, elevated values signify higher confidence. Delving deeper, we find that 94.8%/94.9% of PSMs, 96.2%/96.3% of peptides, and a full 100%/100% of proteins boast probabilities exceeding 99%. Furthermore, the average probabilities stand at 99.4%/99.4% for PSMs, 99.6%/99.5% for peptides, and 99.9%/99.9% for proteins. Shifting focus to the diaPASEF counterparts derived from DIA-NN, we adopted run-specific q-values as the metric for confidence, where a diminished value is synonymous with increased trustworthiness. To illustrate, 100%/100% of precursors and 99.2%/99.1% of protein groups exhibit q-values below 1%. On average, the q-values are 0.00054/0.00054 for precursors and 0.00045/0.00047 for protein groups. To encapsulate, these scores resoundingly demonstrate the high confidence and quality of the identification results within our datasets (Further statistics regarding identification scoring were organized in Supplementary Table 4).

### Proteome quantification

To assess the accuracy and precision of our quantification results, we undertook a detailed quality control analysis. This process involved computing the coefficients of variation (CVs) across technical replicates (Supplementary Fig. 2) and determining the Spearman correlation between these replicates. Diving into the specifics, the two DDA-PASEF datasets revealed median CVs from 0.44% to 0.88% for peptides and from 0.47% to 1.28% for proteins. Furthermore, they exhibited mean Spearman correlations of 0.966/0.974 (IQR: 0.968–0.973/0.972–0.976) at the peptide level and 0.971/0.982 (IQR: 0.961–0.978/0.981–0.984) for proteins. On the other hand, the diaPASEF datasets demonstrated even more impressive patterns, with median CVs from 0.81% to 1.26% for precursors and 0.31% to 0.49% for protein groups. The DIA datasets also showcased striking mean Spearman correlations of 0.989/0.985 (IQR: 0.987–0.991/0.983–0.987) for precursors and an outstanding 0.996/0.995 (IQR: 0.996–0.997/0.995–0.996) at protein group level. These admirably low CVs^40,44^ coupled with the resounding Spearman correlation coefficients attest to the accuracy and robustness of MultiPro in quantitative terms (Detailed statistics and results about CVs and correlation coefficients were displayed in Supplementary Tables 5, 6).

Furthermore, we derived the dynamic range, a measure of quantitative magnitude within a sample, defined by ratio of maximum to minimum values. In the context of our two DDA-PASEF datasets, the median dynamic ranges were 28,558 and 46,758 at the peptide level, and further to 95,896 and 124,277 at the protein level. When examining the DIA-sourced datasets, these metrics expand dramatically to 93,978 and 211,440 at the precursor level and moderate to 11,493 and 34,395 at the protein group tier. Such expansive dynamic ranges underscore the broad quantification horizon of our datasets, fostering more enriched and encompassing identification and quantification (Specific dynamic ranges for each sample and other statistics were also summarized in Supplementary Table 7). Also, to illustrate peptide variability in a quantitative sense, we computed the CVs spanning all samples. Interestingly, when juxtaposed with CVs across technical replicates, these CVs exhibited more than threefold increase across all samples (Supplementary Fig. 3 and Supplementary Table 8). In essence, our datasets not only offer a wide-ranging and holistic view of identification and quantification per sample but also highlight significant variations across samples, while ensuring steadfastness across technical replicates.

### Missing pattern differs by acquisition method, class and batch

**Fig. 2:**
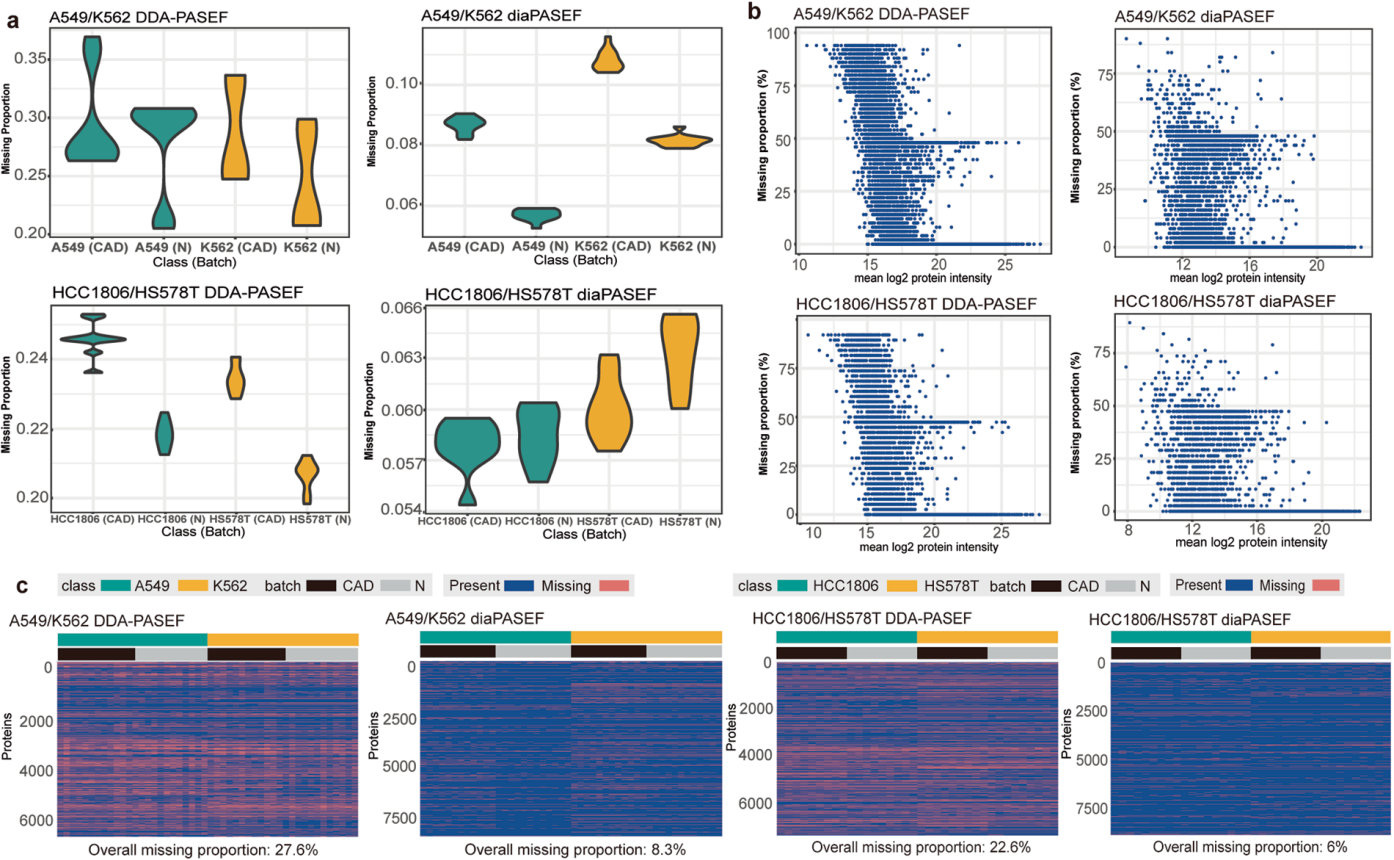
Missing patterns of 4 datasets acquired by DDA-PASEF and diaPASEF methods along A549/K562 and HCC1806/HS578T cell line pairs. (**a**) Violin-plots representing missing proportions of samples categorised by class and batch. (**b**) Scatterplots displaying missing rates of proteins ordered by mean *log*2 intensity. (**c**) Heatmaps demonstrating missing structures and patterns. The classes are differentiated by the coloured bars (A549/HCC1806: green; K562/HS578T: yellow), and the batches are differentiated by shaded bars (CAD: black; N: grey). Missing proteins are coloured in salmon red whereas non-missing proteins are coloured in blue. Here, contamination proteins have been removed in protein matrices.

Various missing structures and mechanisms from different MS methods, classes and batches have been constructed in MultiPro, making it a suitable resource for investigating into missing value issues. We observe that DIA-generated data is consistent in achieving significant improvement over DDA-generated data in terms of overall and sample/protein-wise missing proportions (Supplementary Table 9). Samples quantified by DIA have missing rates from ~5% to ~10% whereas from ~20% to ~35% in DDA datasets (Fig. 2a). Protein missing proportions mainly fall below 50% in DIA datasets and display a uniform distribution from 0% to 100% in DDA counterparts. As missing proportions increase, protein intensities tend to fall with Spearman correlation −0.790, −0.725, −0.507 and −0.461, respectively (Fig. 2b and Supplementary Fig. 4). The overall missingness decreases from more than 20% to ~6% when we transferred the acquisition method from DDA to DIA (Fig. 2c).

Delving into the peptide or precursor levels, we explored the missing structures and discerned analogous trends (Supplementary Table 9). Predominantly, samples quantified via the DIA method exhibited missing rates oscillating between approximately 15% to 30%. In stark contrast, the DDA datasets showcased rates that ranged from about 40% to over 60%. Within the DIA datasets, the missing proportions of peptides or precursors are chiefly below the 50%. Conversely, the DDA datasets presented a more even spread, ranging from 0% to 100%. It’s noteworthy that as protein intensities diminished, the trends in missing proportions increased, correlating at −0.697, −0.678, −0.551, and −0.563 respectively (Supplementary Fig. 5). Emphasizing the broader picture, the shift in acquisition from DDA to DIA remarkably reduced the overall missingness rate from roughly 50% to a mere 20%.

Beyond the interplay between abundance and the protein missing rate, we also investigated the relationship between identification score and missing proportion, unearthing discernible patterns therein. Specifically, within the two DDA-derived datasets, where a higher score denotes heightened confidence, the Spearman correlation coefficients that relate identification score to the missing proportion stand at −0.530 and −0.512 for peptides, and −0.360 and −0.337 for proteins (Supplementary Figs. 6, 7). And in the context of the diaPASEF datasets, where a lower score is synonymous with elevated confidence, the correlations ascend to 0.705 and 0.696 at the precursor level and 0.665 and 0.609 at the protein group tier (Supplementary Figs. 6, 7). In summation, a superior score potentially signifies a diminished missing rate, with the DIA paradigm manifesting a more pronounced relationship in this regard than DDA.

Finally, in our datasets, missing proportions also vary according to the classes and batches. Different cell lines demonstrate various missing rate ranges and missing structures (Fig. 2a,c), and when comparing the missingness between batches, batch N displays lower proportion of missing values as compared to batch CAD, except for the HCC1806/HS578T diaPASEF dataset (Fig. 2a, bottom right). Therefore, the missing patterns in our datasets can also provide useful scenarios for evaluating the performance of MVI methods when missing values interact with batch or class effects.

### Mixed effects from class, batch and acquisition method

**Fig. 3:**
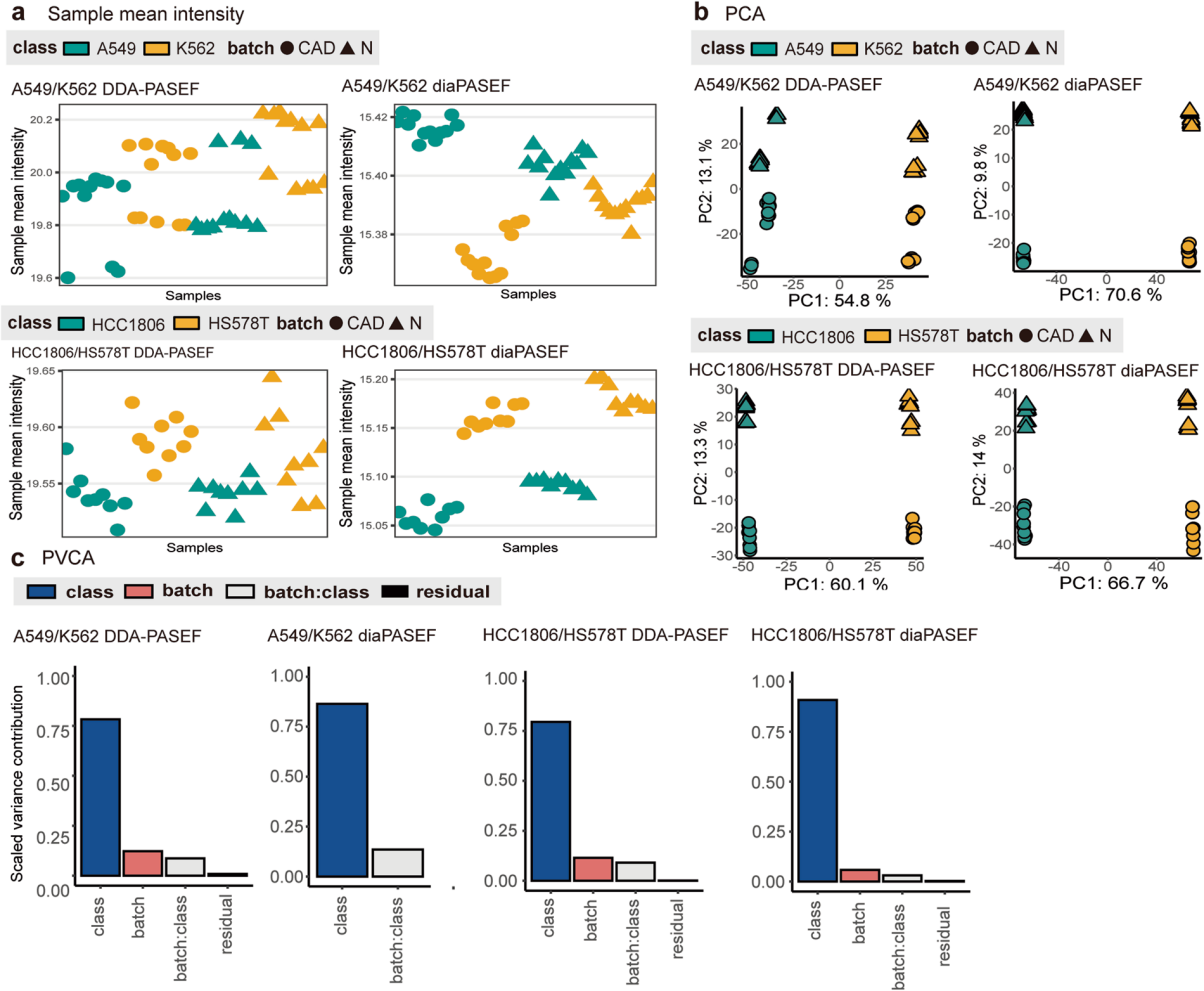
Batch effects diagnosis across A549/K562 and HCC1806/HS578T cell line datasets acquired by DDA-PASEF and diaPASEF. (**a**) Scatterplots demonstrating the mean intensities of samples. (**b**) Principal Component Analysis visualization of MultiPro. The colours represent the class (green for A549/HCC1806 and yellow for K562/HS578T). The shapes represent the batch (circle: CAD and triangle: N). (**c**) Principal Variance Component Analysis of MultiPro. Each coloured bar represents the source of variation (blue: class effects, red: batch effects, grey: batch and class interaction effects and black: residual effects).

Development of powerful BECAs requires well-designed batch-affected datasets for benchmarking. Using different MS machines, mixed effects from class, batch and acquisition method have been established in our datasets, which can be investigated using visual and quantitative tools recommended in proBatch^7^, including sample mean intensities, Principal Component Analysis (PCA)^45,46^ and Principal Variance Component Analysis (PVCA)^47^ (Fig. 3).

In MultiPro, class effects were demonstrated to be the predominant source of variation, with prevalent batch effects. The shifts in sample mean intensities are mostly contributed by class (differentiated by colours), while the batches introduce slight shifts in intensities (differentiated by shapes) (Fig. 3a). Similarly, the PCA scatterplots display class-driven sample clusters along the first principal component (PC1, greatest source of variation), while clusters led by batch effects can also be observed along PC2 (Fig. 3b). PVCA reports similar findings (Fig. 3c), although it is unable to detect the batch term in the A549/K562 diaPASEF dataset which evidently contains batch effects, as observed in the sample mean intensities and PCA scatterplot (Fig. 3a,b). Lastly, as contrast with DDA, DIA always leads to more robust quantification results with fewer drifts, slighter fluctuations and higher proportion of class effects companied by lower batch proportion (Fig. 3).

## Usage Notes

### Missing value imputation benchmarking

Here, we provide a case study to demonstrate the application of MultiPro for benchmarking of various MVI methods. Several commonly used MVI algorithms were selected, including mean imputation, median imputation, K-nearest neighbours (KNN)^12^, probabilistic minimum (MinProb)^48^, local least squares (LLS)^49^ and quantile regression imputation of left-censored data (QRILC, https://cran.r-project.org/web/packages/imputeLCMD/). And HCC1806/HS578T DDA-PASEF and diaPASEF datasets were utilized to compare the efficacy of these MVI methods by simulation. In detail, for the protein matrices, increasing total proportions (10%, 20%, 30%, 40% and 50%) of missing values were generated in missing not at random (MNAR) and missing completely at random (MCAR) modes with ratio of 3:1. Normalized root mean squared error (NRMSE) was then used as a metric to evaluate the differences between the imputed and original data.

**Fig. 4:**
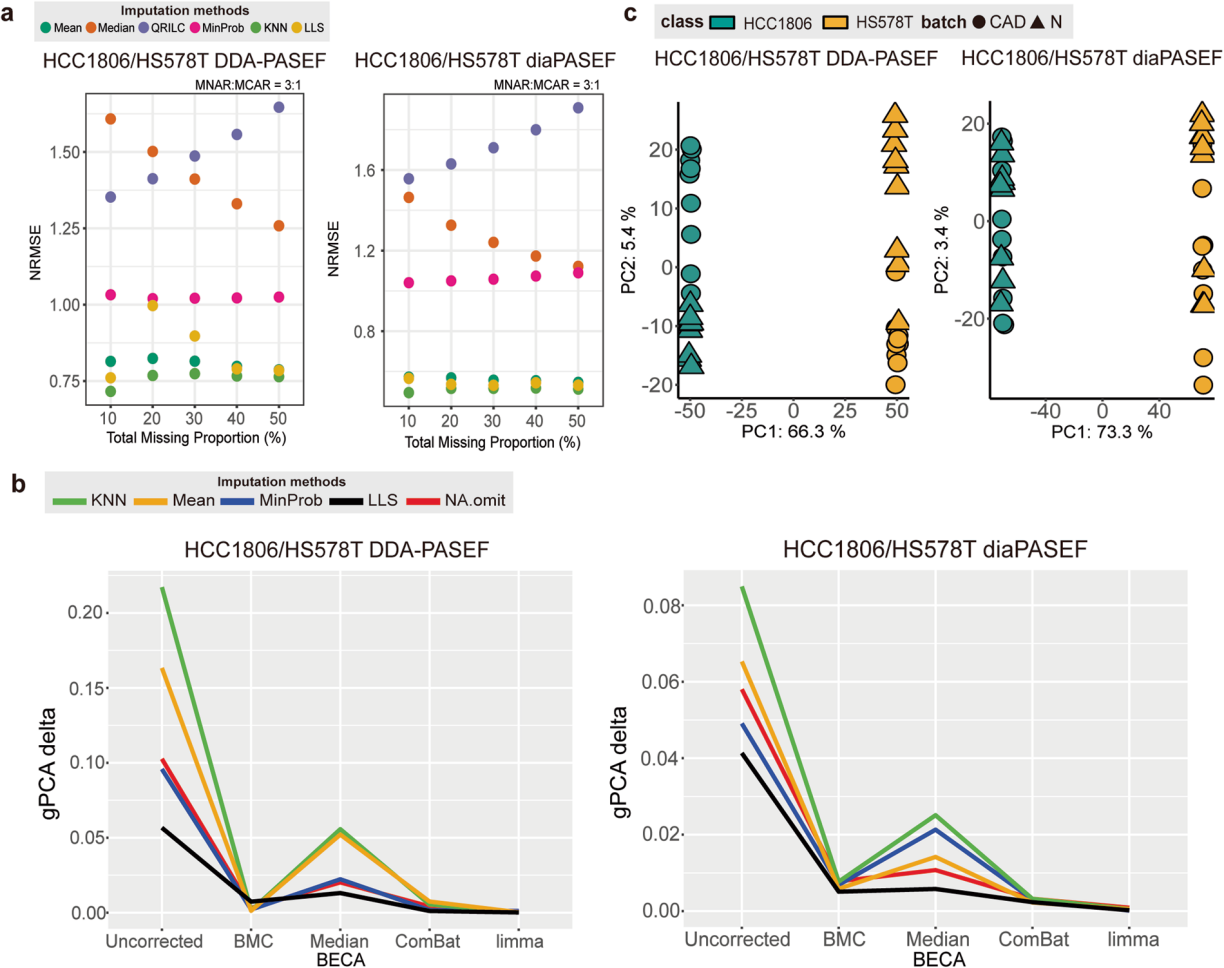
Case studies based on HCC1806/HS578T DDA-PASEF and diaPASEF datasets. (**a**) Scatterplot representing NRMSE values derived from simulations, in order of increasing total missing proportion (10, 20, 30, 40, 50%). Missing values were simulated in 3:1 ratio of MNAR to MCAR. The imputation methods used here include mean, median, QRILC, MinProb, KNN and LLS and are differentiated by the colours of dots. (**b**) Line plot demonstrating gPCA delta values after different combinations of MVI and BEC methods. The imputation methods are differentiated by the colour of the lines (green: KNN, yellow: mean, blue: MinProb, black: LLS, and red: NA.omit). The batch correction methods are displayed on the x-axis (uncorrected, BMC, median centering, ComBat and limma). (**c**) PCA scatterplot of ComBat batch-corrected datasets. Classes are differentiated by the colours (green: HCC1806 and yellow: HS578T), and batches are differentiated by the shapes (circles: CAD and triangles: N).

According to Fig. 4a, our comparisons report KNN imputation as the best method among all the imputation algorithms, across all the missing proportions. Most of the MVI methods remain stable with increasing total MV proportions. However, QRILC imputation does not seem to perform well, with a significant increase in NRMSE values as missing proportions increase. Lastly, DDA has different benchmarking patterns with DIA, and top-performance MVI methods can achieve better NRMSE in DIA datasets than in DDA.

### Batch effect correction benchmarking

Using the HCC1806/HS578T DDA-PASEF and diaPASEF datasets, we compared the level of batch effects after different combinations of MVI methods and BECAs. Specifically, KNN, Mean, MinProb, LLS and NA.omit (dropping proteins containing missing value) were selected as 5 different ways of dealing with missing values, in combination with 4 different BECAs (batch mean centering (BMC), median centering, ComBat^13^, limma^50^) and no correction as a control. The gPCA delta metric was used for batch effect level evaluation^51^.

Based on our experiments (Fig. 4b), ComBat, limma, median centering and BMC result in significant reduction of batch effects compared to the uncorrected data. Generally, the LLS imputation outperforms other imputation methods across different batch correction strategies rather than previous KNN imputation. In contrast with DDA, DIA displays slight differences in benchmark rankings with a smaller batch effect size.

We also did post-correction diagnosis of batch effects based on ComBat-corrected datasets (Fig. 4c). The variation explained by PC2 (the batch PC) has decreased a lot, indicating significant reductions in batch effects after correction. However, slight batch effects can still be observed in PCA plots, meaning that more powerful BECA needs to be developed for thoroughly resolving batch issues.

### Supplementary information

Supplementary Figures

Supplementary Tables

## Data Availability

The R scripts for reproducing the main figures are available through the GitHub repository at https://github.com/kaipengl/batcheffectsdataset.
