# Supplementary material for: MultiPro: DDA-PASEF and diaPASEF acquired cell line proteomic datasets with deliberate batch effects
Source: Sci Data. 2023 Dec 2;10:858. doi: 10.1038/s41597-023-02779-8 (PMC10693559; doi:10.1038/s41597-023-02779-8)
Supplement: Supplementary file 1 — Supplementary Figures [file 41597_2023_2779_MOESM1_ESM.pdf]

Table of contents

Supplementary Fig. 1: Protein coverage distribution. 2

Supplementary Fig. 2: Coefficients of variation across technical replicates. 4

Supplementary Fig. 3: Peptide variability measured by coefficients of variation across all samples. 5

Supplementary Fig. 4: Relationship between missing proportion and abundance of protein (DDA) or protein group (DIA) measured by Spearman correlation coefficients......... 6

Supplementary Fig. 5: Relationship between missing proportion and abundance of peptide (DDA) or precursor (DIA) measured by Spearman correlation coefficients. 7

Supplementary Fig. 6: Relationship between missing proportion and score of peptide (DDA) or precursor (DIA) measured by Spearman correlation coefficients......... 8

Supplementary Fig. 7: Relationship between missing proportion and score of protein (DDA) or protein group (DIA) measured by Spearman correlation coefficients. 9

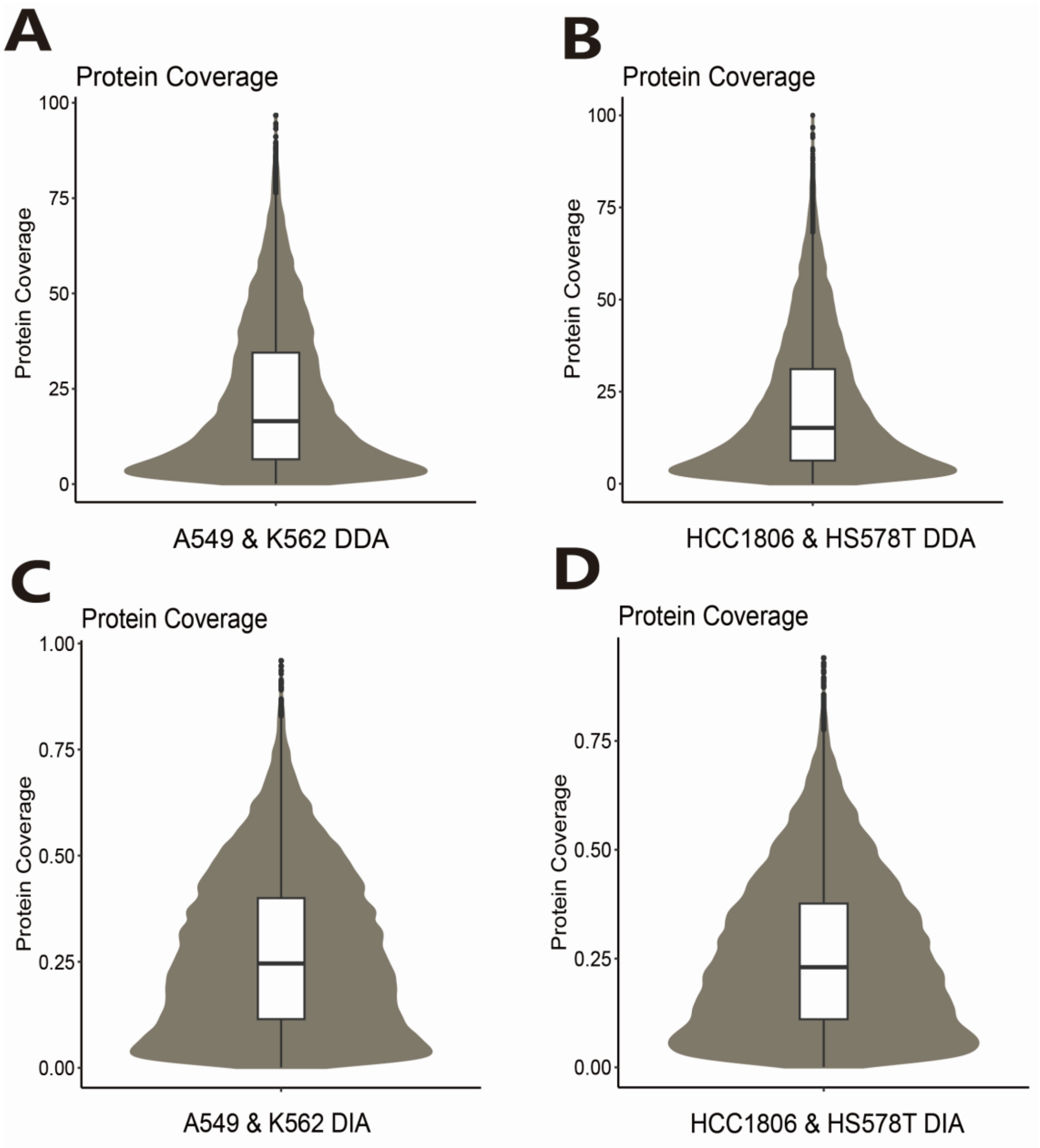

Supplementary Fig. 1: Protein coverage distribution of (A) A549 & K562 DDA dataset; (B) HCC1806 & HS578T DDA dataset; (C) A549 & K562 DIA dataset; (D) HCC1806 & HS578T DIA dataset.

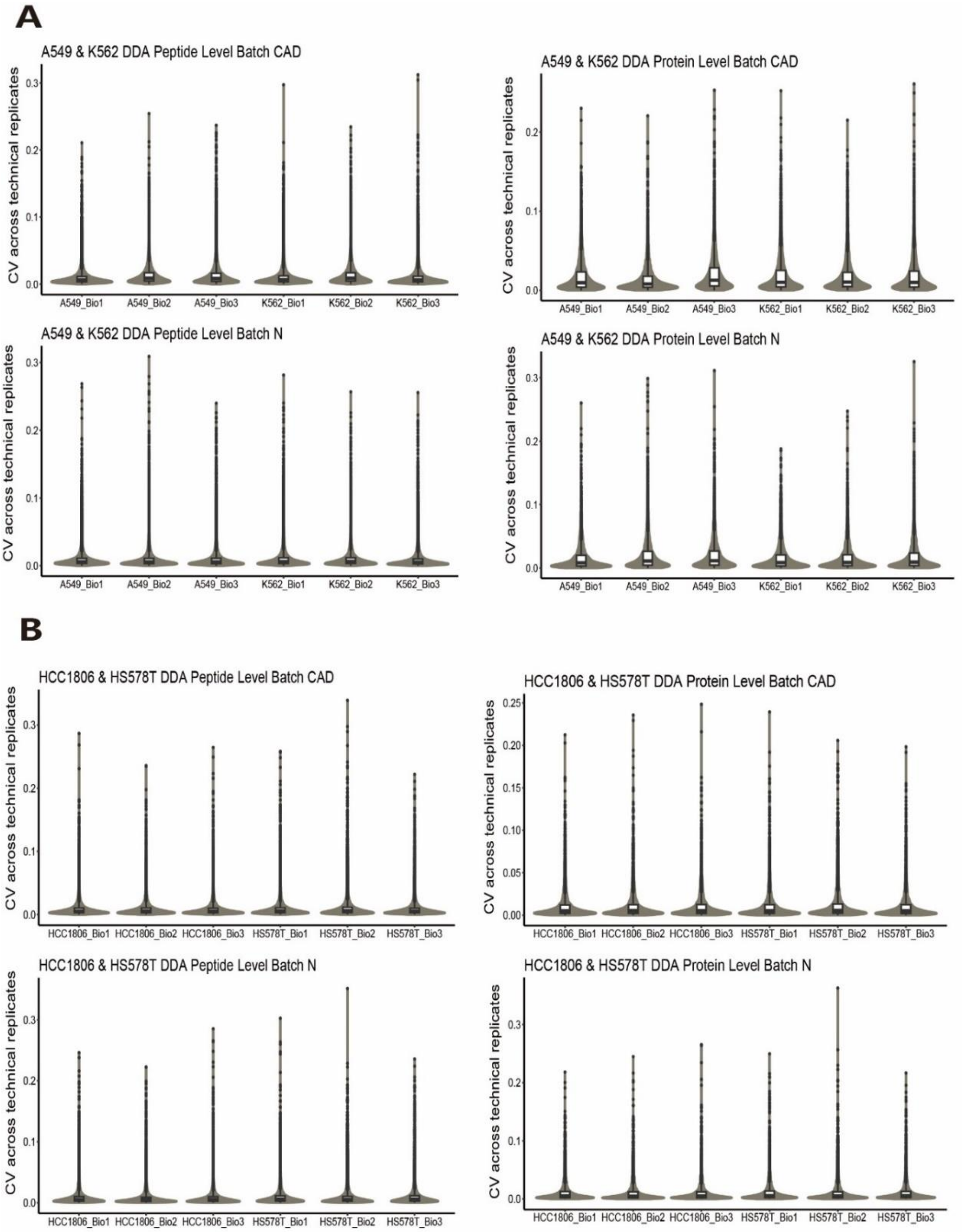
A
A549 & K562 DDA Peptide Level Batch CAD
A549 & K562 DDA Protein Level Batch CAD
A549 & K562 DDA Peptide Level Batch N
A549 & K562 DDA Protein Level Batch N
CV across technical replicates
A549_Bio1
A549_Bio2
A549_Bio3
K562_Bio1
K562_Bio2
K562_Bio3
B
HCC1806 & HS578T DDA Peptide Level Batch CAD
HCC1806 & HS578T DDA Protein Level Batch CAD
HCC1806 & HS578T DDA Peptide Level Batch N
HCC1806 & HS578T DDA Protein Level Batch N
HCC1806_Bio1
HCC1806_Bio2
HCC1806_Bio3
HS578T_Bio1
HS578T_Bio2
HS578T_Bio3

(DIA).

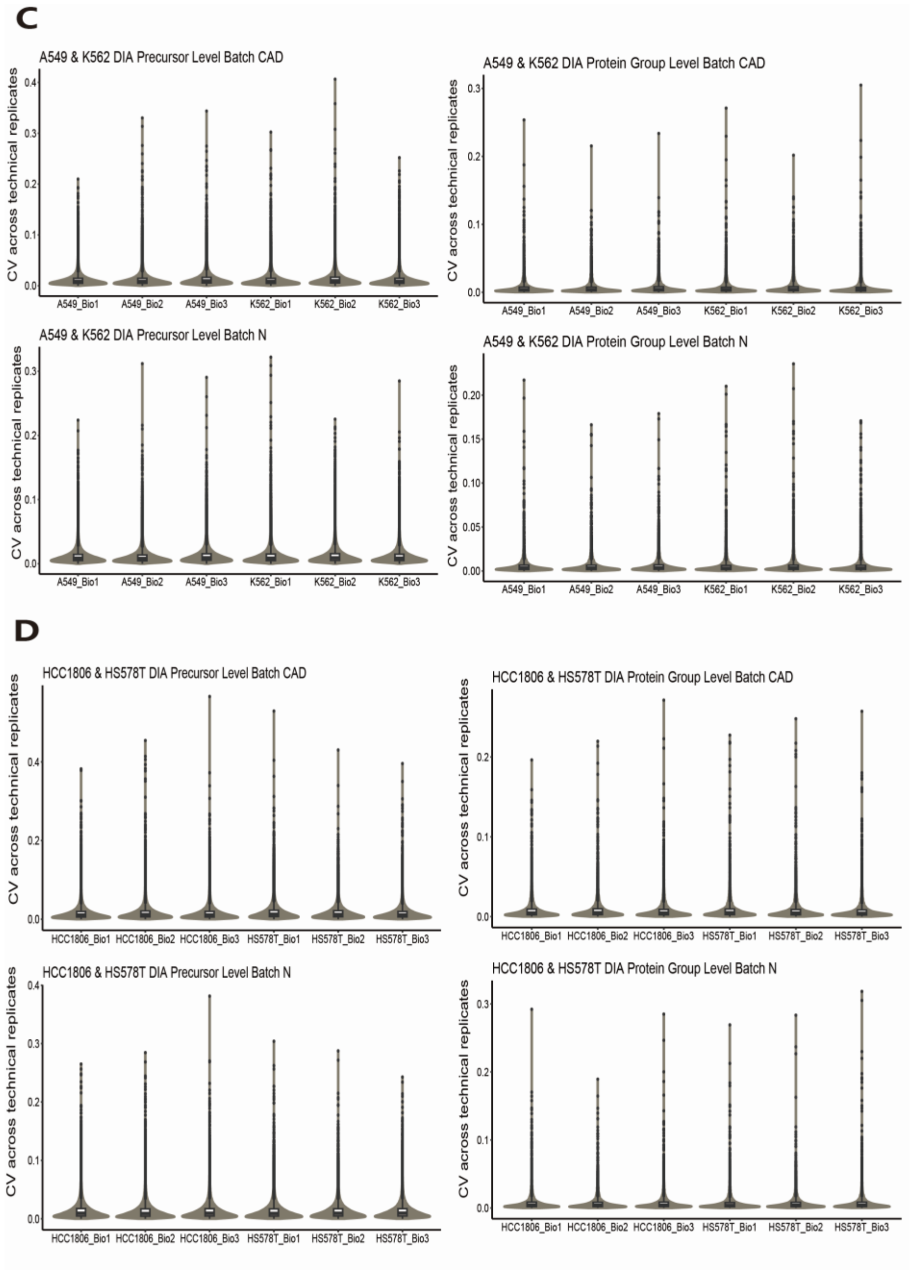

Supplementary Fig. 2: Coefficients of variation across technical replicates from (A) A549 & K562 DDA dataset; (B) HCC1806 & HS578T DDA dataset; (C) A549 & K562 DIA dataset; (D) HCC1806 & HS578T DIA dataset at 1) peptide and protein level (DDA); 2) precursor and protein group level (DIA).

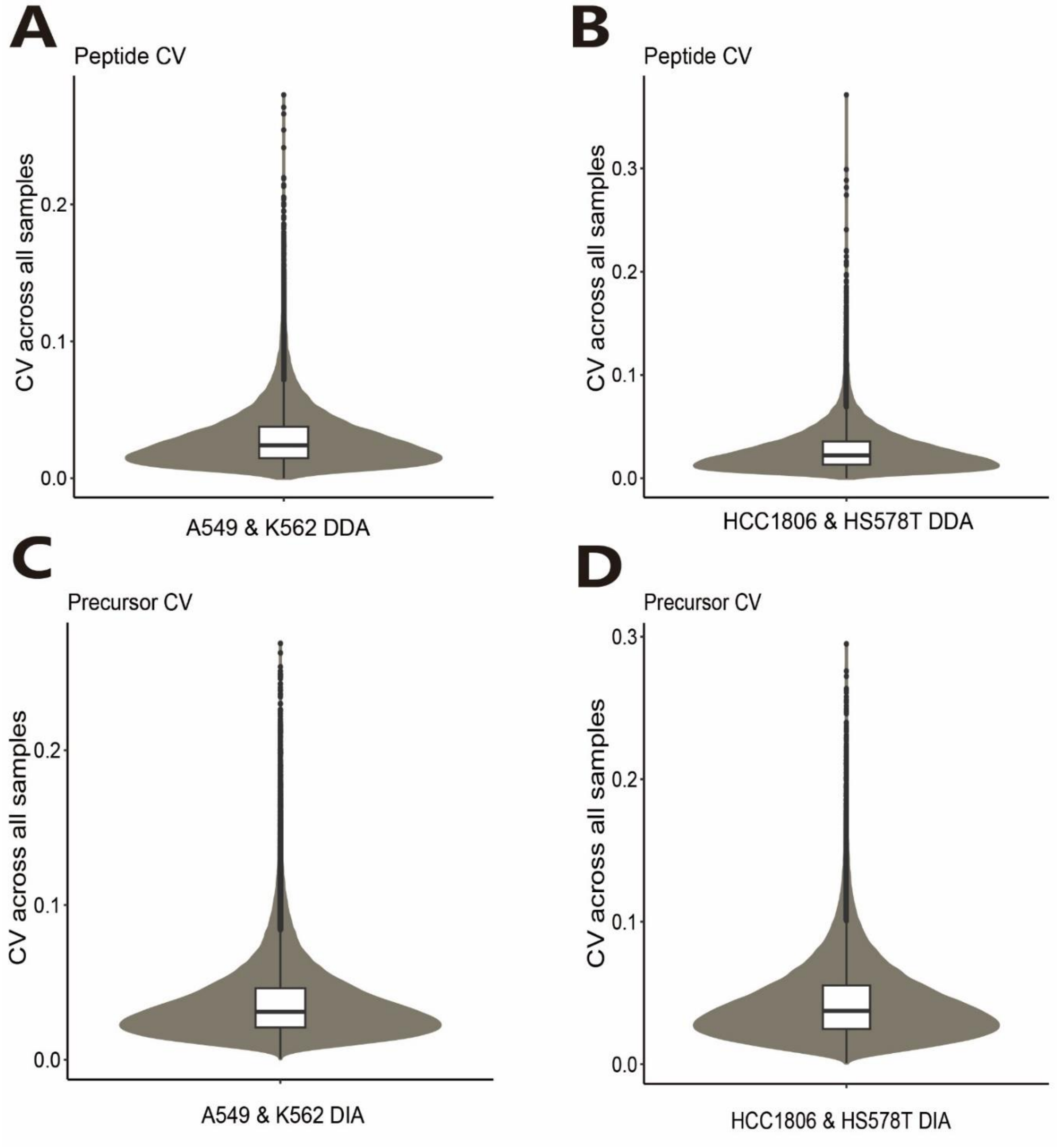

Supplementary Fig. 3: Peptide variability of (A) A549 & K562 DDA dataset; (B) HCC1806 & HS578T DDA dataset; (C) A549 & K562 DIA dataset; (D) HCC1806 & HS578T DIA dataset measured by coefficients of variation across all samples.

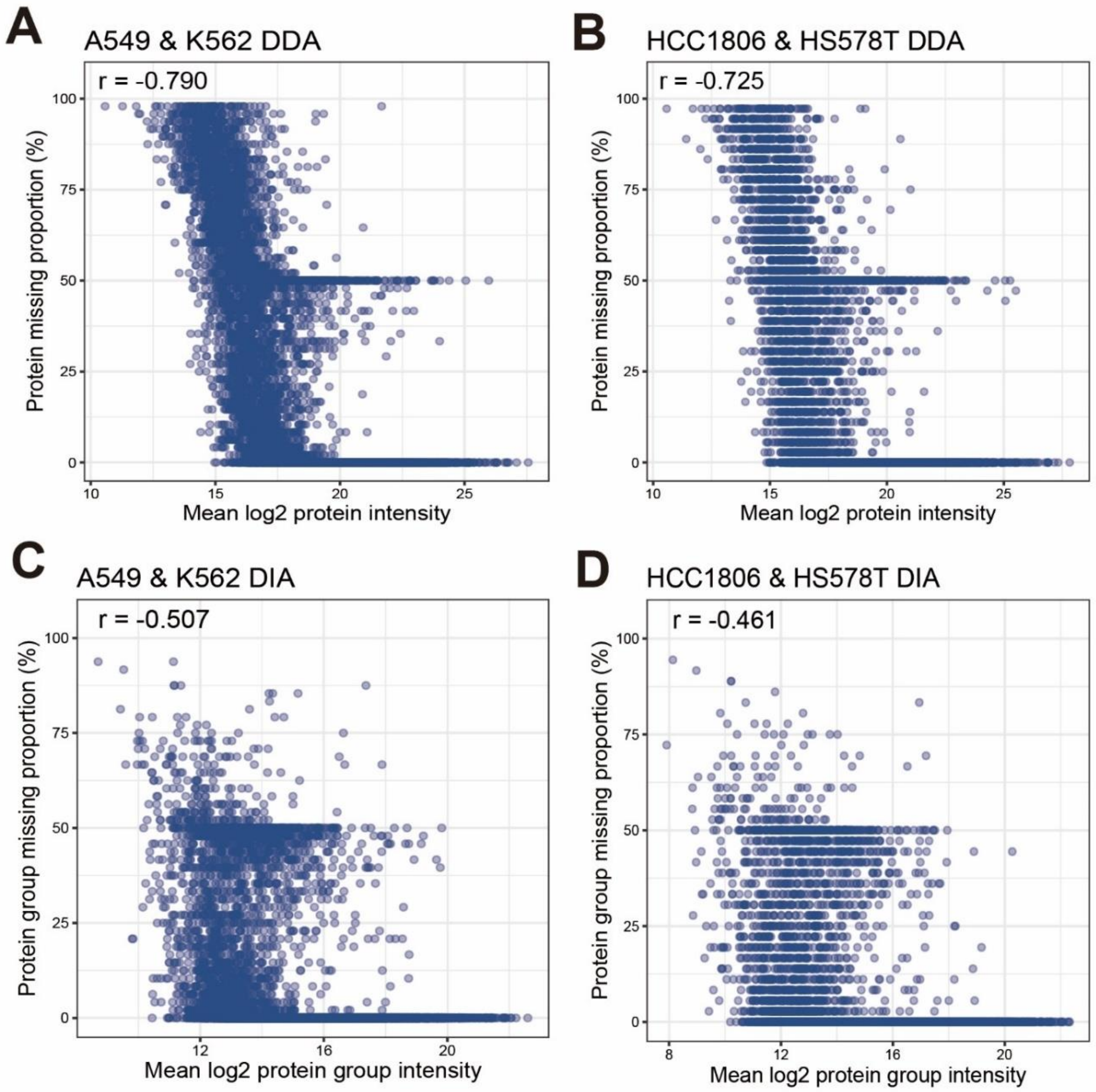

Supplementary Fig. 4: Relationship between missing proportion and abundance of protein (DDA) or protein group (DIA) from (A) A549 & K562 DDA dataset; (B) HCC1806 & HS578T DDA dataset; (C) A549 & K562 DIA dataset; (D) HCC1806 & HS578T DIA dataset measured by Spearman correlation coefficients.

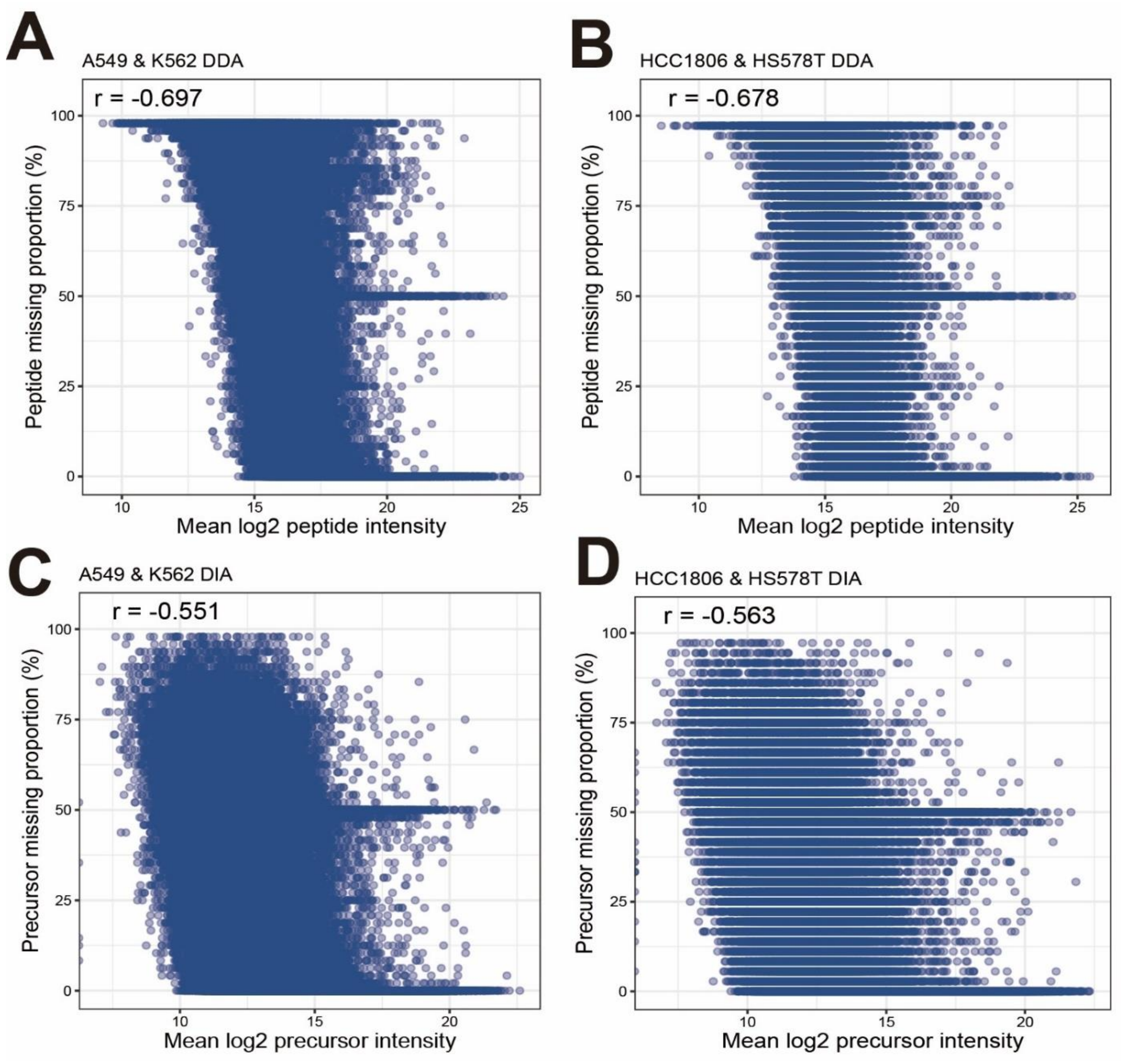

Supplementary Fig. 5: Relationship between missing proportion and abundance of peptide (DDA) or precursor (DIA) from (A) A549 & K562 DDA dataset; (B) HCC1806 & HS578T DDA dataset; (C) A549 & K562 DIA dataset; (D) HCC1806 & HS578T DIA dataset measured by Spearman correlation coefficients.

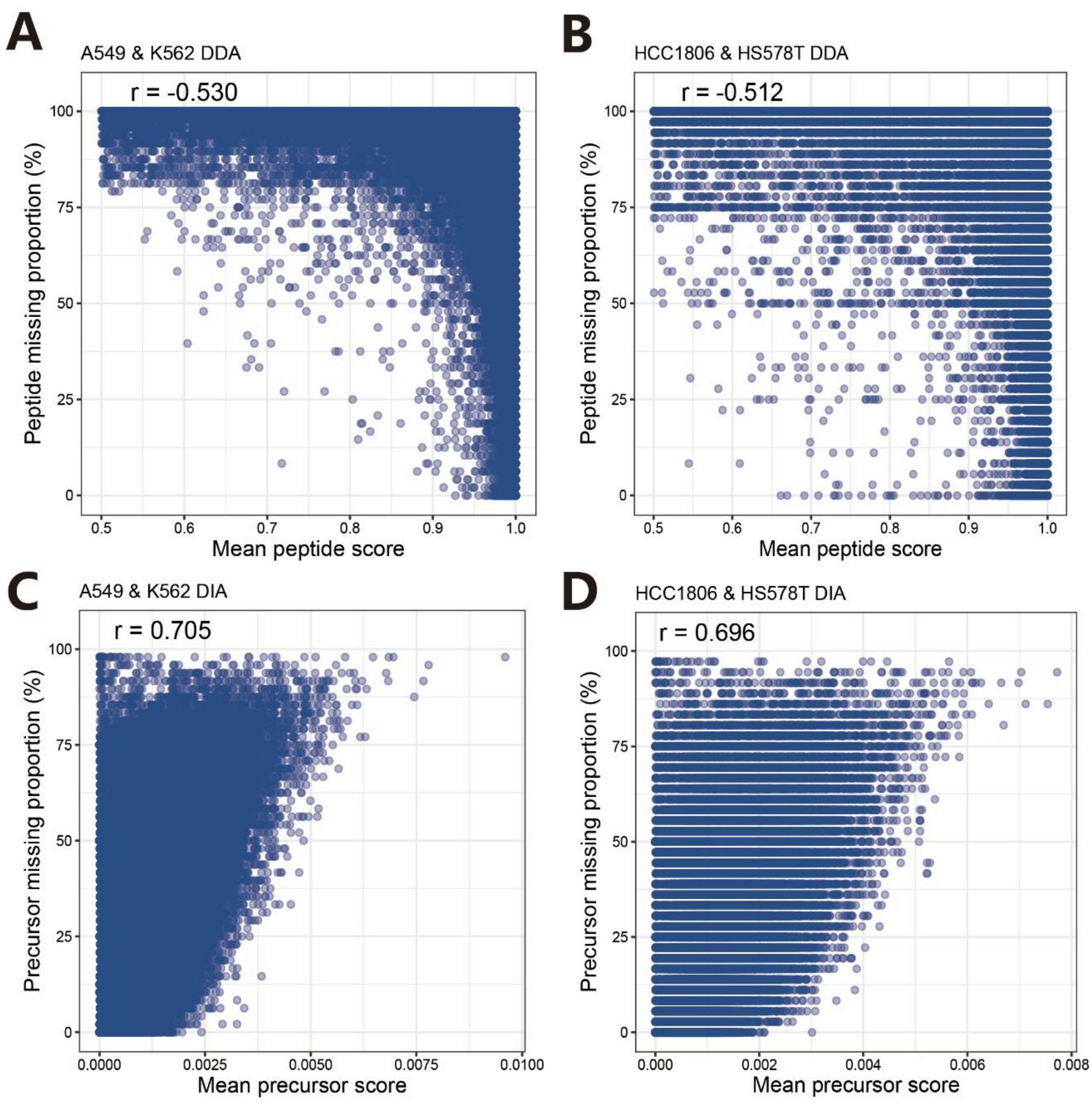

Supplementary Fig. 6: Relationship between missing proportion and score of peptide (DDA) or precursor (DIA) from (A) A549 & K562 DDA dataset; (B) HCC1806 & HS578T DDA dataset; (C) A549 & K562 DIA dataset; (D) HCC1806 & HS578T DIA dataset measured by Spearman correlation coefficients.

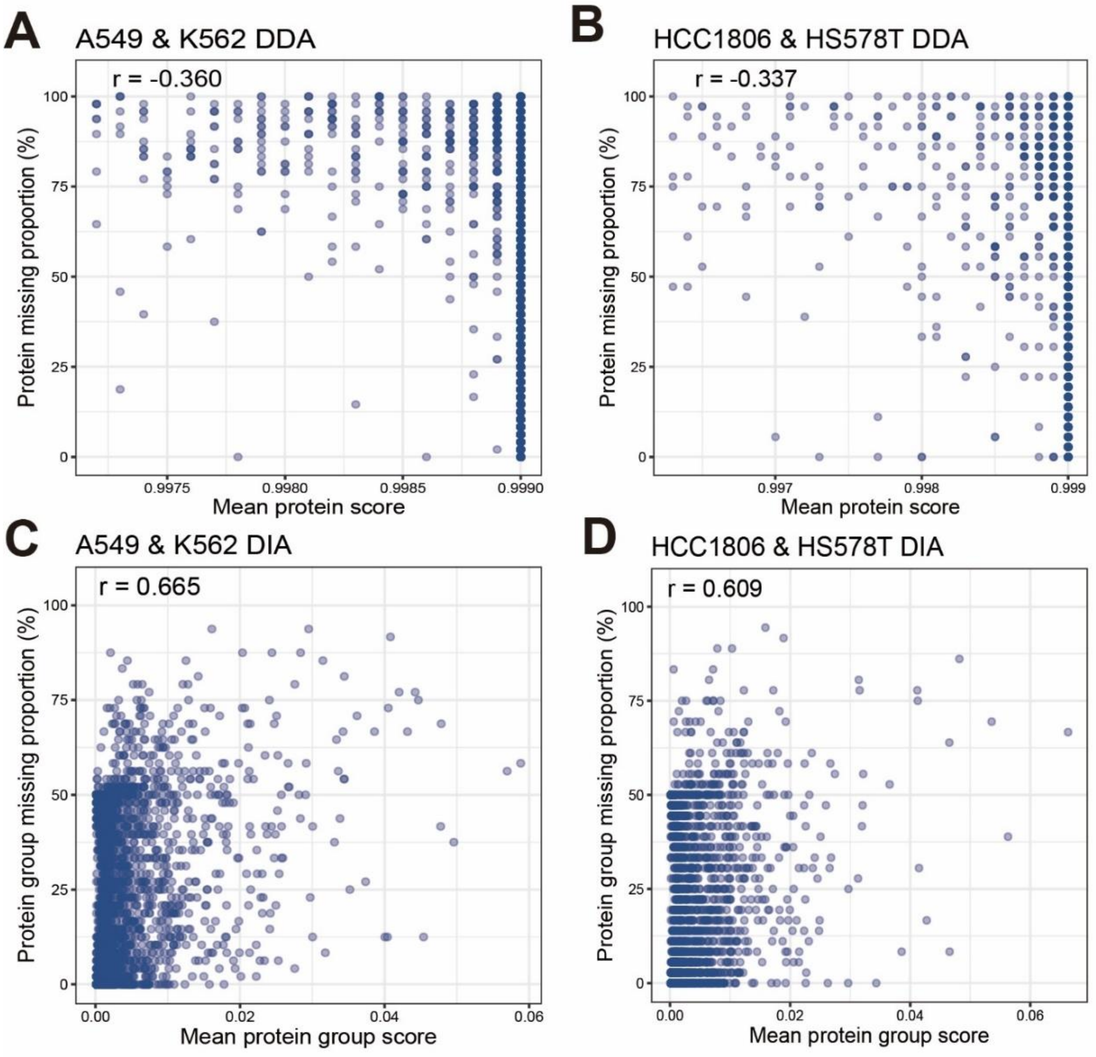

Supplementary Fig. 7: Relationship between missing proportion and score of protein (DDA) or protein group (DIA) from (A) A549 & K562 DDA dataset; (B) HCC1806 & HS578T DDA dataset; (C) A549 & K562 DIA dataset; (D) HCC1806 & HS578T DIA dataset measured by Spearman correlation coefficients.
